# News and Miscellany

**Published:** 1905-09

**Authors:** 


### News and Miscellany.

NOT A CASE OF YELLOW FEVER on Texas soil at the present writing.

ANY ONE having numbers 2, 5, and 6, Vol. 16, 1859 issue of the *New Orleans Medical and Surgical Journal,* will confer a great favor by communicating with that journal. P. O. Drawer 602, New Orleans, La.

DR. C. H. WILKINSON, who has been in Western Texas for the past two years perfecting himself in the study of "Tuberculosis," has returned to Galveston, his old home, where he will resume practice and reside in the future.

THE NEW UNITED STATES PHARMACOPEIA now recognizes and incorporates amongst the official preparations Listerine (*Liquor Antisepticus*), Antiphlogistine (*Emplastrum Kaolinæ*), and Fellows' Comp. Syrup Hypophosphites (*Syrup Hypohosphites Comp.*).

KASAGRA (Stearn's) is an elegant preparation. It is a combination of Cascara Sagrada and aromatics. It is about the only thing I know that will cure constipation. It does not purge or disturb the stomach. Tastes like peppermint candy.

IN THE MATTER OF USING proprietary medicines advertised to physicians only. If there is a practicing physician in Texas who does not use and never has used them I want his name. I will send him the "Red Back" free. Their use is universal. As well try to stem the current of the Mississippi as to try to stop it.

"AGIN" THE LAW.—A private telegram to the "Red Back" from Inspector Dr. L. W. Cook, in charge of a Texas detention camp says that a Mrs. Meyer was shot and killed while attempting to evade quarantine; Mrs. Stego Meyer, of New Orleans. She was trying to get into Texas by the (hot) Air-Line.

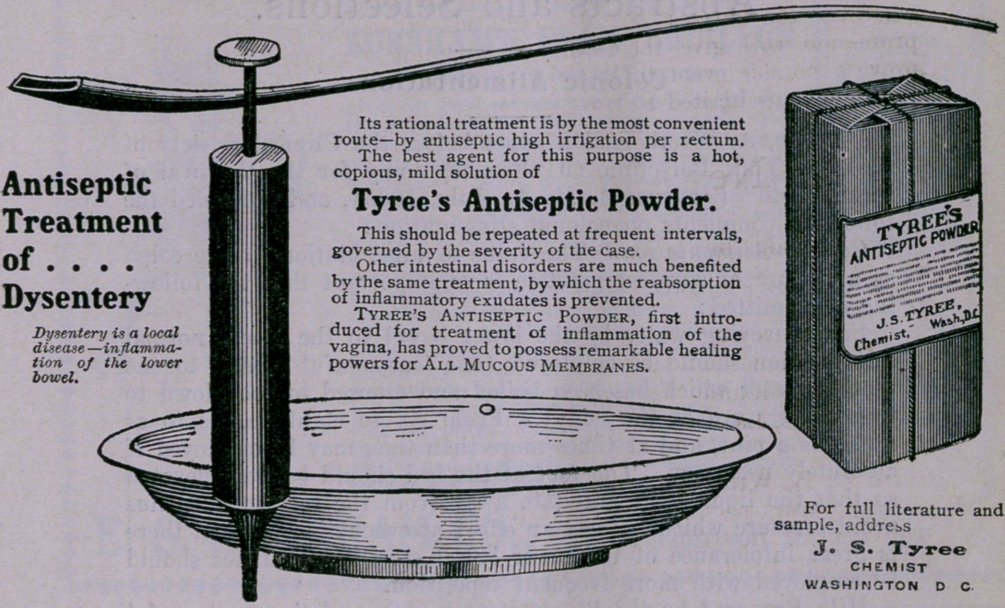

KILLED A PATIENT.—At the North Texas Hospital for the Insane a violent patient by the name of Dr. Jackson was found dead with ribs broken and other evidence of brutal treatment. Three of the attendants were arrested charged with the killing, and after a hearing were admitted to bail. Dr. J. S. Turner, the Superintendent, promptly investigated the case and caused the arrest of the parties. He will prosecute them vigorously.

---

FOR SALE.—On account of wife's death, I will sell for cash, or part cash, seven-acre lot with first class new residence, six rooms, good out improvements, fine orchard, good water, a twelve years' location. Collections good. Address: Dr. R. E. Grigg, Ad Hall, Milam county, Texas.

---

DR. R. H. L. BIBB, of Saltillo, Mexico, so well and favorably known to the medical profession of Texas, we regret to learn, has resigned the position of Chief Surgeon of the Consolidated System of Mexican Railroads.

---

DR. T. H. HARRELL, formerly of Hutto, Texas, but now of Peublo, Mexico, is surgeon to the Mexican National Railroad at that place, and has charge of the big new Mexican National Railroad hospital. A separate building for consumptives is being erected by the company.

